# Jumping mechanism in the marsh beetles (Coleoptera: Scirtidae)

**DOI:** 10.1038/s41598-022-20119-5

**Published:** 2022-09-22

**Authors:** Konstantin Nadein, Alexander Kovalev, Stanislav N. Gorb

**Affiliations:** Functional Morphology and Biomechanics, Zoological Institute, Christian-Albrechts University of Kiel, Am Botanischen Garten 1-9, 24118 Kiel, Germany

**Keywords:** Physiology, Zoology

## Abstract

The jumping mechanism with supporting morphology and kinematics is described in the marsh beetle *Scirtes hemisphaericus* (Coleoptera: Scirtidae). In marsh beetles, the jump is performed by the hind legs by the rapid extension of the hind tibia. The kinematic parameters of the jump are: 139–1536 m s^−2^ (acceleration), 0.4–1.9 m s^−1^ (velocity), 2.7–8.4 ms (time to take-off), 0.2–5.4 × 10^–6^ J (kinetic energy) and 14–156 (g-force). The power output of a jumping leg during the jumping movement is 3.5 × 10^3^ to 9.6 × 10^3^ W kg^−1^. A resilin-bearing elastic extensor ligament is considered to be the structure that accumulates the elastic strain energy. The functional model of the jumping involving an active latching mechanism is proposed. The latching mechanism is represented by the conical projection of the tibial flexor sclerite inserted into the corresponding socket of the tibial base. Unlocking is triggered by the contraction of flexor muscle pulling the tibial flexor sclerite backwards which in turn comes out of the socket. According to the kinematic parameters, the time of full extension of the hind tibia, and the value of the jumping leg power output, this jumping mechanism is supposed to be latch-mediated spring actuation using the contribution of elastically stored strain energy.

## Introduction

Terrestrial arthropods utilize various ways of locomotion among which jumping is rather widespread. Jump allows animals to reach their prey, avoid predators or simply shorten distances. The majority of arthropods are capable to perform jumps or jump-like movements by their limbs, mouthparts or by other body parts^1,2^. Some of them are specialized jumpers with developed jumping apparatus. There are especially many of them among insects, where even two entire orders, fleas (Siphonaptera)^3,4^ and orthopterans^5–11^, evolved as specialized jumpers. A large number of hemipterans like leafhoppers (Cicadellidae)^12,13^, froghoppers (Cercopoidea)^14,15^, planthoppers (Fulgoridae)^16^, shore bugs (Heteroptera: Saldidae)^17^ are known as capable jumpers. Jumping performance and mechanisms have been investigated also for the stick insects (Phasmatodea: Timematidae)^18^, snow fleas (Mecopera: Boreidae)^19^, lacewings (Neuropetra: Chrysopidae)^20^, caddies flies (Trichoptera)^21^, parasitoid wasps (Hymenoptera: Pteromalidae, Braconidae, Figitidae, Ichneumonidae)^22^, and scorpion flies (Mecoptera: Panorpidae)^23^.

The largest order of insects, beetles (Coleoptera), is known for the fact that its representatives have the widest range of abilities for locomotion. Beetles are capable of walking, running, flying, swimming, digging, and jumping. The latter originated repeatedly and independently in the evolution of different groups of beetles and was accompanied by corresponding morphological specializations. Jumping beetles are known from such families as leaf beetles (Chrysomelidae, subfamilies Galerucinae and Bruchinae), weevils (Curculionidae, subfamilies Curculioninae, Ceutorhynchinae, Erirhininae), jewel beetles (Buprestidae, subfamilies Agrilinae, Trachyinae), marsh beetles (Scirtidae)^24^ and, recently, the jumping mechanism in flea beetles and weevils has been experimentally studied^25,26^. Jump in both of these beetle families are characterized by the use of swollen hind legs with well-developed muscles. Excepting marsh beetles, they also share the presence of a sclerotized and enlarged metafemoral extensor tendon (MET) as a key element of their jumping apparatus^27^.

**Figure 1:**
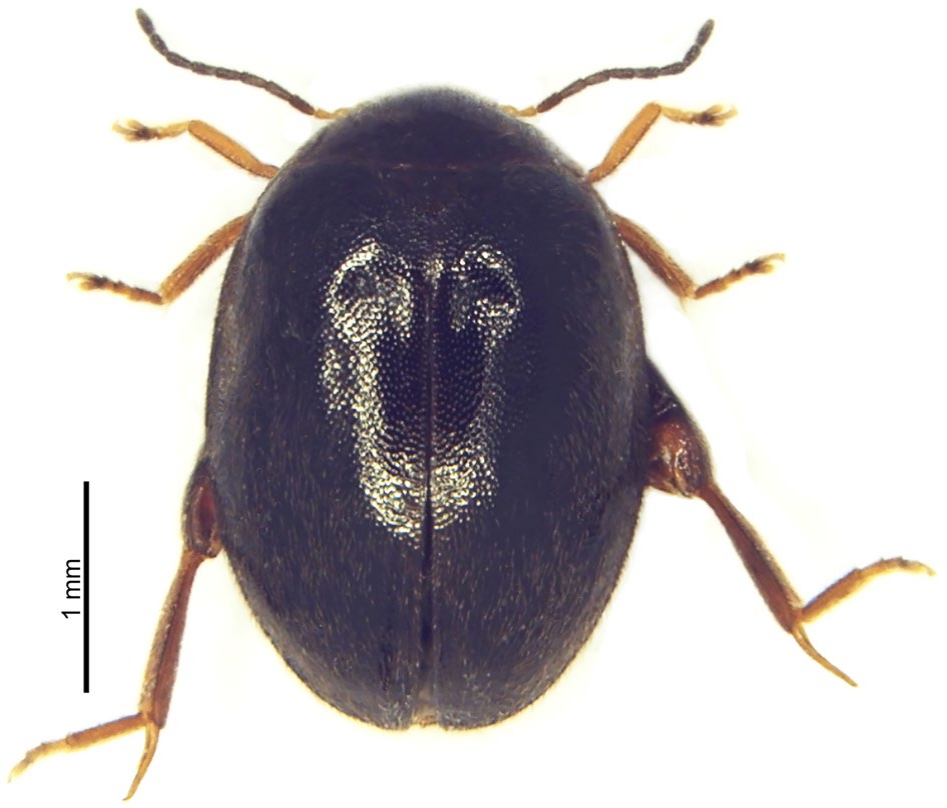
General appearance of the marsh beetle *Scirtes hemisphaericus* (Coleoptera: Scirtidae).

Marsh beetles Scirtidae is a cosmopolitan family with more than 60 genera and about 1600 species described so far^28^. Imagoes inhabit wet habitats as diverse as ponds and rivers shores, swamps and flood-meadows and feed on decomposed plant materials and flowers, often occurring openly on plants. Two genera, *Scirtes* Illiger, 1807 and *Ora* Clark, 1865, are known for their ability to fast jumps. Marsh beetles use hind legs with swollen metafemora for jumping, but lack sclerotized metafemoral extensor tendon^27^ that is morphologically fundamentally different from other jumping beetles, which in turn may suggest a different jumping mechanism. However, until recently, the jumping mechanism and its kinematic parameters in Scirtidae remained unexplored. This article aims to fill this gap by studying details of the jumping mechanism in the marsh beetle *Scirtes hemisphaericus* (Fig. 1) and to demonstrate the diversity of the biomechanical solutions for similar evolutionary challenges.

## Results

### Structure of the jumping legs

**Figure 2:**
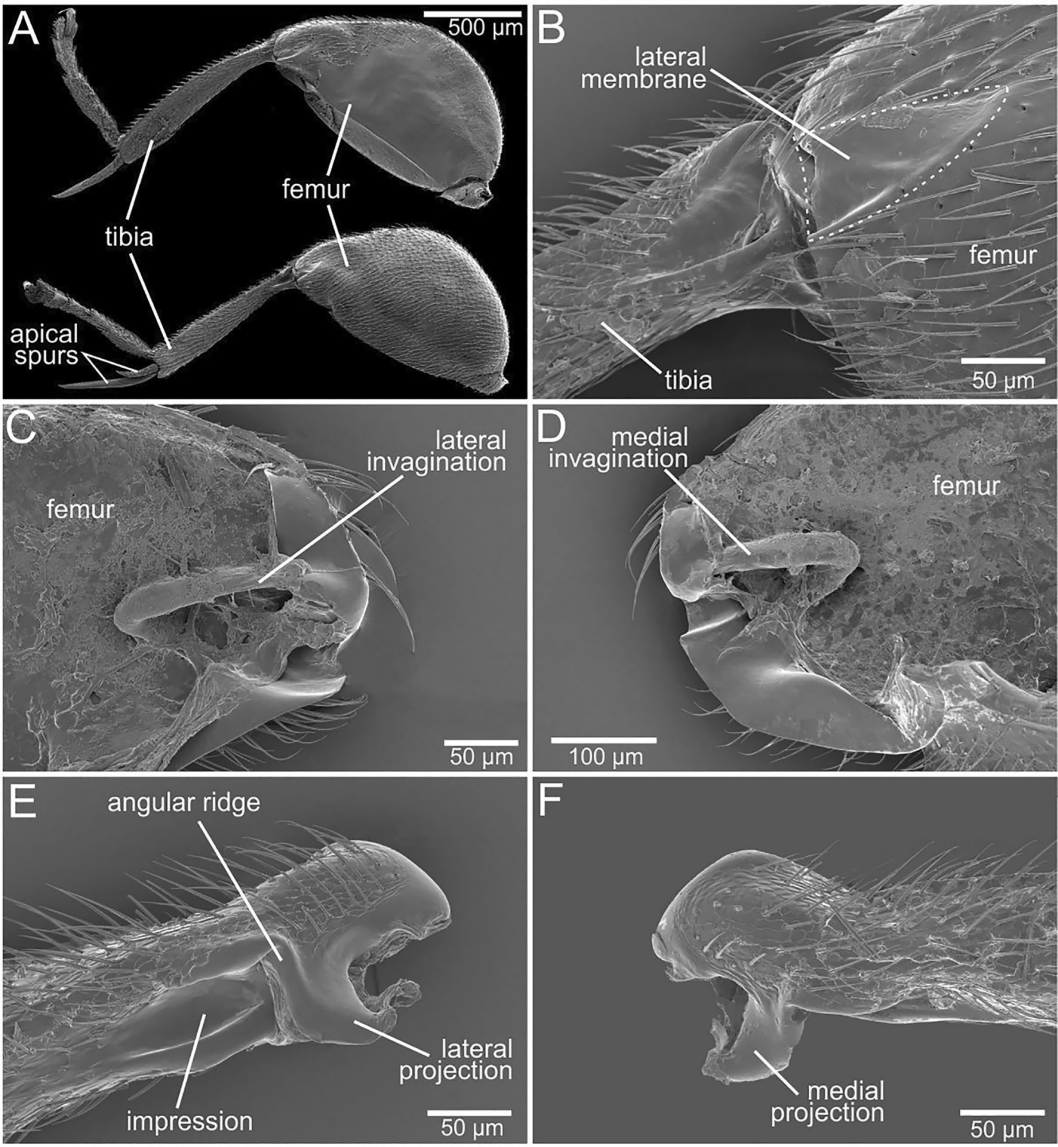
Structure of the jumping legs and details of the femoro-tibial joint of *Scirtes hemisphaericus*, scanning electron microscopy (SEM). (**A**) Hind (jumping) leg, general appearance, upper image—the medial side; bottom image—the lateral side. (**B**) Femoro-tibial joint, lateral external view, lateral membrane is outlined by dashed line. (**C**,**D**) Femur in the region of femoro-tibial joint, sagittal section. (**E**,**F**) Tibial base, antero-lateral view.

**Figure 3:**
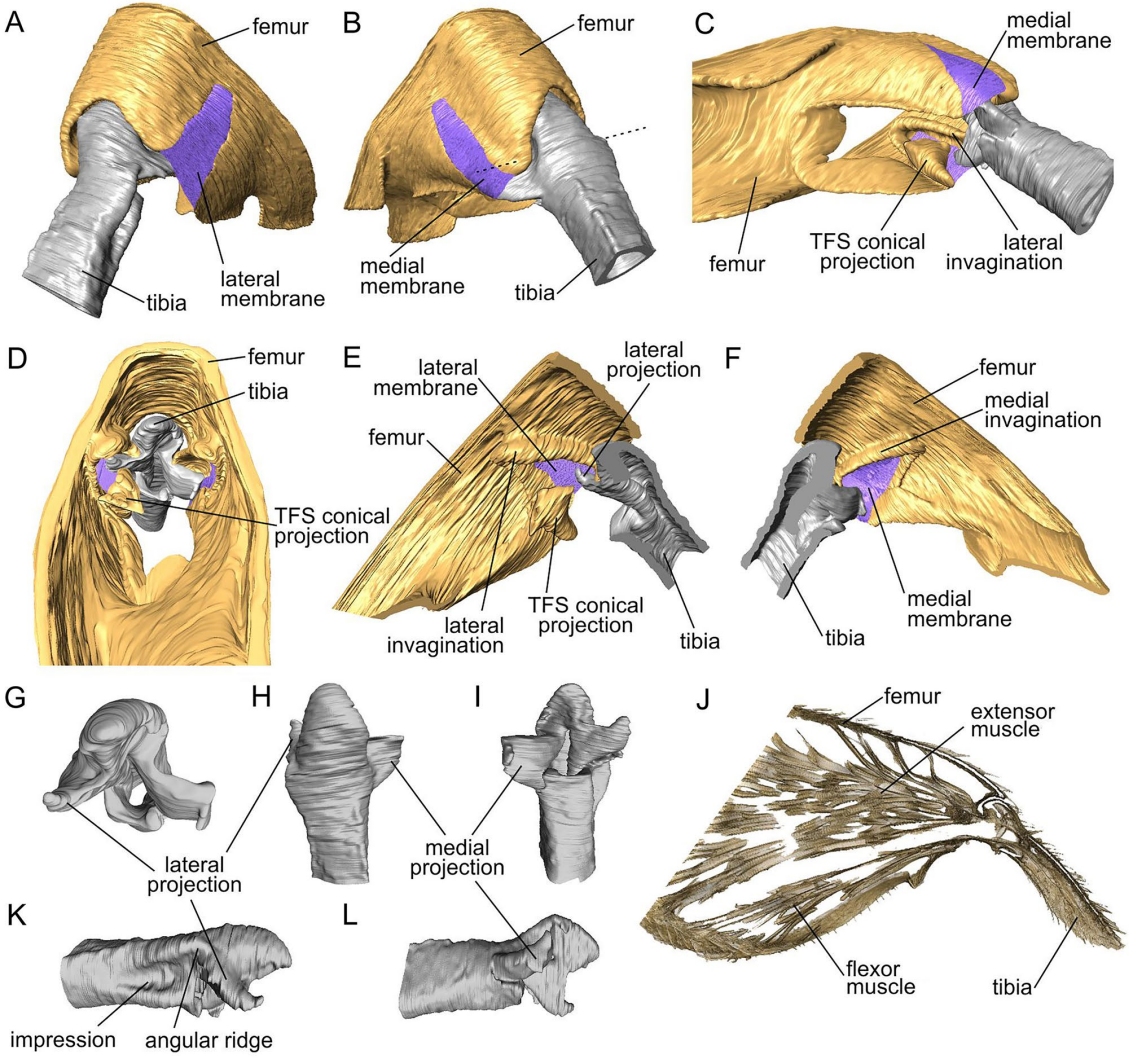
Femoro-tibial joint of the hind jumping leg of *Scirtes hemisphaericus*, synchrotron X-ray micro-computed tomography (SR-µCT). (**A**,**B**) Femoro-tibial joint, antero-lateral view, dashed line shows the axis of rotation. (**C**) Femoro-tibial joint, ventral view. (**D**) Femur, transversal section proximally to the femoro-tibial joint. (**E**,**F**) Femoro-tibal joint, sagittal section. (**G**–**I**,**K**,**L**) Tibial base: (**G**) posterior view; (**H**) dorsal view; (**I**) ventral view; (**K**,**L**) lateral view. (**J**) Muscular system of the femur, SR-µCT data volume reconstruction, semi-thin sagittal section. *TFS* tibial flexor sclerite.

The hind (jumping) leg (Figs. 2, 3) is characterised by the swollen femur with the ratio length-to-width of about 1.8 (Fig. 2A). Its ventrolateral side bears moderately deep longitudinal depression serving as a socket for the fully flexed tibia. The femoro-tibial joint (Figs. 2B, 3A–F) has rather complex structure of its counterparts. The cuticle of the femoral counterpart of the joint in the most apical part bears an invagination on each side. Lateral invagination is situated outermost from the central body line of the beetle (Figs. 2C, 3C,E) while the medial invagination (Figs. 2D, 3F) is situated closer to the central body line of the beetle. The invagination is of an elongate-triangular shape (resembling “>”) with proximally-oriented acute apex and with swollen margins projecting into the femoral cavity. The dorsal side of margins is larger, thicker and projecting further inside the femoral cavity. The invaginations are externally covered by the membranous cuticle of a triangular shape, called here lateral membrane (Figs. 2B, 3A) and medial membrane (Fig. 3B), respectively.

**Figure 4:**
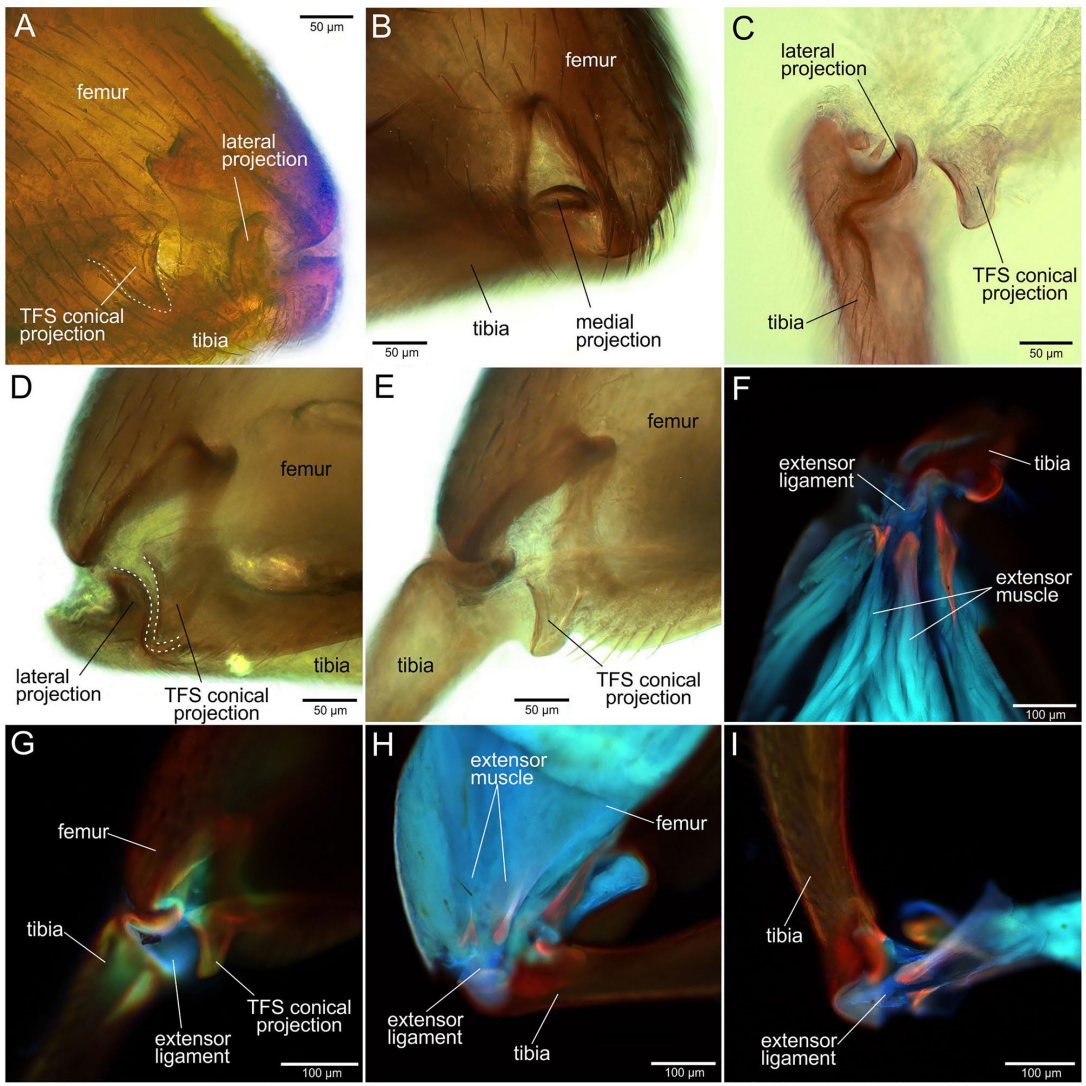
Femoro-tibial joint of the hind jumping leg of *Scirtes hemisphaericus*, light microscopy (**A**–**E**) and confocal laser scanning microscopy (CLSM) (**F**–**I**). Resilin-bearing extensor ligament is bright blue or purple-blue. (**A**) Femoro-tibial joint with flexed tibia showing the position of lateral projection, external view, lateral side. (**B**) Femoro-tibial joint with flexed tibia showing the position of medial projection, external view, medial side. (**C**) Tibial base and tibial flexor sclerite with the conical projection of tibial flexor sclerite (TFS). (**D**) Femoro-tibial joint with the flexed tibia, TFS conical projection inserted into the socket of tibial base and is in locked position. TFS conical projection and corresponding socket on tibia are outlined by white dashed line. (**E**) Femoro-tibial joint with extended tibia, external view; TFS conical projection is outside of the corresponded socket of tibial base and is in unlocked position. (**F**) Tibial base and muscles connected by the extensor ligament, lateral view. (**G**) Femoro-tibial joint externally, lateral view. (**H**) Sagittal section of the femur. I, Tibial base with extensor ligament, lateral view. *TFS* tibial flexor sclerite.

The tibia is nearly as long as femur (Fig. 2A), slightly curved, when viewed from above and possesses a pair of long apical spurs, one of which is 2.2 times shorter than tibia and the other one is about twice shorter than the first one. The tibial counterpart of the joint is represented by the tibial base with a pair of asymmetric projections on each side (Figs. 2E,F, 3I–L). The lateral projection (Figs. 2E, 3G,H,K, 4A) is shorter and inserts into the lateral invagination of the femur (Fig. 3D,E) at the flexed position of the tibia, while the medial projection (Figs. 2F, 3H,I,L, 4B) is longer inserting into medial one, respectively (Fig. 3D,F). The lateral side of the tibia, right behind the lateral projection, possesses a deep elongated ‘impression’ with the prominent ‘angular ridge’ situated on the side of the lateral projection (Figs. 2E, 3K).

Tibial flexor sclerite (TFS) is asymmetric and conditionally consists of two undivided parts, ‘basal plate’ and ‘conical projection’, respectively (Figs. 3C–E, 4C–E). The ‘basal plate’ of the TFS is more or less elongate-triangular plate, attached distally by the broader edge to the tibial base and proximally by the narrower apex to the flexor muscles. The ‘conical projection’ is its enlarged, sclerotized lateral side of conical shape situated at the lateral invagination (Figs. 3C–E, 4C). When the tibia is flexed, the ‘conical projection’ is pushed deeper in the femur and changed its orientation so that it inserts into ‘impression’ and fits into the ‘angular ridge’ behind the lateral projection of the tibia (Fig. 4D). When the tibia is extended, the ‘conical projection’ extends from the femoral cavity, its tip protrudes outward and it becomes partially visible in the side view (Fig. 4E). Muscular system of the hind leg consists of extensor muscle and flexor muscle (Fig. 3J). The volume of the extensor muscle is at least twice larger than that of the flexor (Fig. 3J). The extensor muscle is connected to the tibial base by the extensor ligament (Fig. 4F–I). The extensor ligament contains resilin, which presence is supported by the data of the confocal laser scanning microscopy and highlighted blue in the Fig. 4F–I.

### Jumping performance, kinematic parameters and energy output of the jumping leg

**Figure 5:**
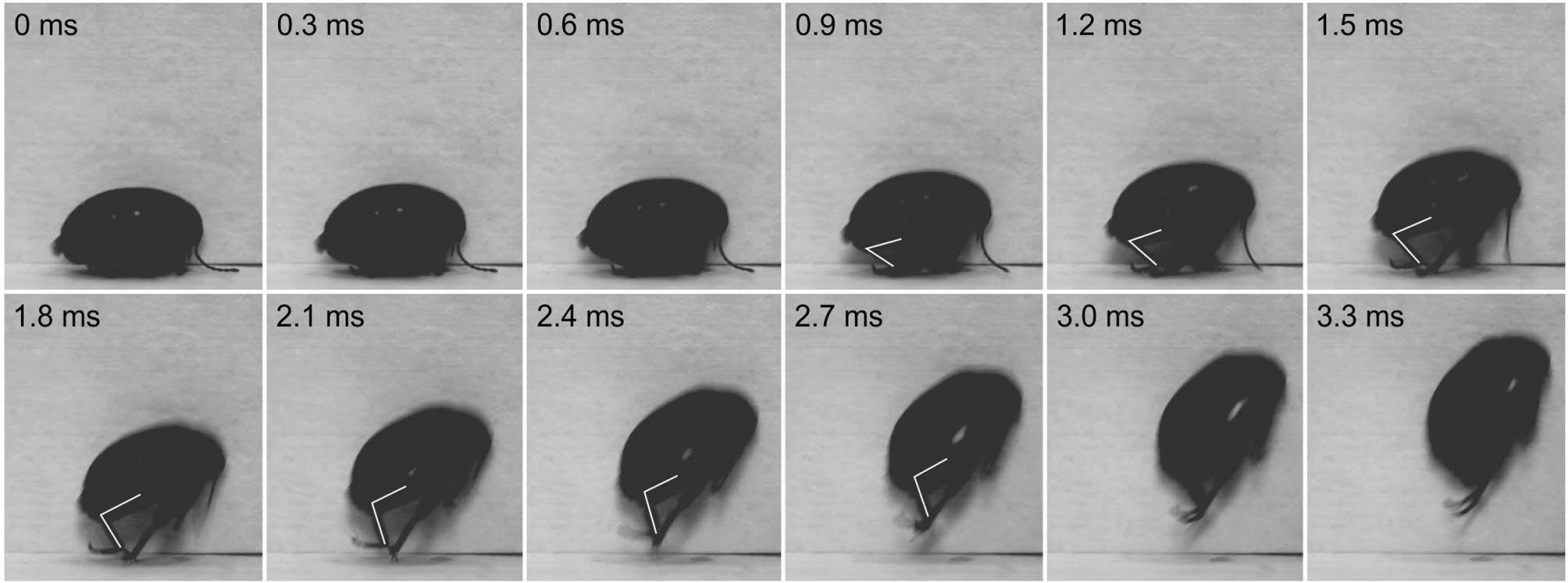
Frame-by-frame depiction of the jump of the marsh beetle *Scirtes hemisphaericus*. Jump is elicited by two hind legs, 3000 frames s^−1^. The position of the left hind leg is indicated by the white line.

**Figure 6:**
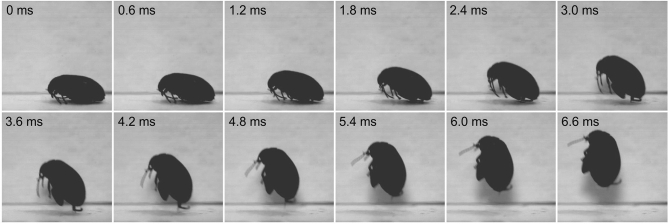
Frame-by-frame depiction of the jump of the marsh beetle *Scirtes hemisphaericus*. Jump is elicited by one hind legs, 3000 frames s^−1^.

**Figure 7:**
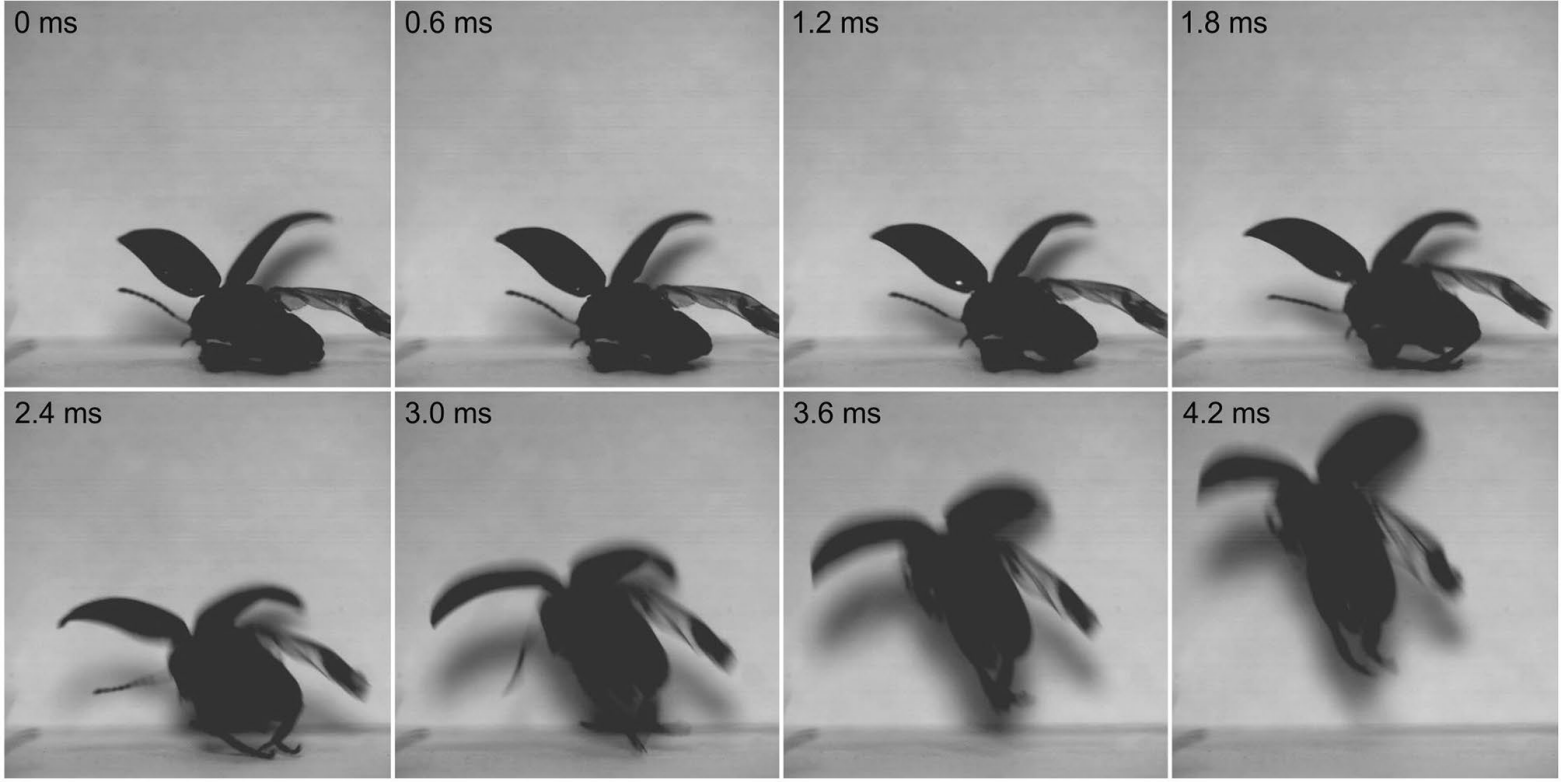
Frame-by-frame depiction of the jump of the marsh beetle *Scirtes hemisphaericus*. Jump is elicited by two hind legs with opened wings, 3000 frames s^−1^. The initial stage with wings opening (about 42 ms) is omitted.

The jump is performed by the hind legs as supported by the high-speed videography analysis (Figs. 5, 6, 7, SI Movies 1–3). The key act of the jump is a rapid extension of the tibia from the fully flexed position to the fully extended one. The process of jumping consists of several consecutive steps. (1) The body is usually in the horizontal position with fully flexed hind tibiae (0–0.6 ms) (Fig. 5, SI Movie 1). (2) The extension of hind tibiae with simultaneous lifting up the body; hind tarsi and apical spurs are in full contact with the substrate (0.9–1.5 ms) (Fig. 5). (3) Further lifting up the body, which orientation to substrate becoming closer to vertical; the hind legs are touching the substrate by apical spurs only (1.8–2.4 ms) (Fig. 5). (4) Take-off, the body is completely airborne, the hind tibiae are fully extended (2.7 ms and further) (Fig. 5). In a short time after the take-off a beetle’s body may experience a rotation in various directions as well as change its orientation.

The movement of both jumping legs is asynchronous that evidenced from the observation of one-leg jumping (Fig. 6, SI Movie 2). This mode of jumping is not rare (4 cases of 25 recorded). Another mode is jumping with opened wings (Fig. 7, SI Movie 3). The process of jumping is the same as for two-leg mode, but it is preceded by the wing opening that takes much longer than jump itself. Hind wings can be fully or partially opened at the moment of take-off. The hind wings may start to flap before the take-off or remain inactive during the process of jumping. Jumping with opened wings is also common (9 cases of 25 recorded). As evidenced from the observations in the nature and in the laboratory, the beetles always sitting on the substrate with the fully flexed tibiae are ready to jump immediately and such jump is often passed to the flight.

Kinematics of jumping is summarized in Tables 1 and 2. The differences in kinematic parameters in various modes of jumping are noticeable (Table 2). The highest score of parameters, i.e. the shortest take-off time and the highest acceleration and velocity, is attributable for jumping by two legs, while those for the jumping with opened wings are on an average lower. The jumping by one leg has an intermediate score of parameters.

**Table 1:** Kinematic parameters of jumping in the marsh beetle *Scirtes hemisphaericus* (Coleoptera: Scirtidae) in comparison with other jumping insects, average values in brackets. Mode of jumping: *s* spring-actuated mechanism, *d* direct muscle contraction.

Name	Mode	Take-off time (ms)	Velocity (m s^−1^)	Acceleration (m s ^−2^)	Kinetic energy ( × 10^–6^ J)	g-force	References
Marsh beetle *Scirtes hemisphaericus* (Coleoptera: Scirtidae)	s	2.7–8.4 (4.1)	0.4–1.9 (1.3)	139–1536 (743)	0.2–5.4 (2.9)	14–156 (75)	Present paper
Weevil *Orchestes fagi* (Coleoptera: Curculionidae: Curculioninae)	s	1.5–3.0 (2.2)	0.7–2.0 (1.3)	530–1965 (1048)	0.3–4.4 (2.2)	54–200 (106)	^26^
Flea beetles (Coleoptera: Chrysomelidae)	s	1.1–7.7	0.7–2.9	100–2660	0.9–19.7	83–342	^25,61^
Locusts (Orthoptera)	s	–	3.2	180	9–11	–	^6^
Fleas Siphonaptera	s	1.2–1.6	1.1–1.9	727–1600	0.4–1.8	75–160	^4^
Leafhoppers (Hemiptera: Cicadellidae)	s	2.3–6.4	1.1–2.9	188–1055	0.6–77	19–225	^12^
Froghoppers (Hemiptera: Cercopoidea)	s	0.8–1.5	2.5–4.7	1667–5400	28–238	170–550	^14^
Planthoppers (Hemiptera: Issidae)	s	0.7–1.6	2.2–5.5	1295–7051	75–303	133–719	^16^
Shore bugs (Hemiptera: Saldidae)	s	3.4–3.9	1.3–1.8	335–529	3.4	34–54	^17^
Pygmy mole crickets (Orthoptera: Tridactylidae)	s	1.8–3.3	3.2–5.4	1043–3000	54–124	106–306	^32^
Bush crickets (Orthoptera: Tettigoniidae)	d	21.0–32.6	1.0–2.12	83.4–143.8	125–1380	–	^8^
Caddis flies (Trichoptera)	d	14.5–17	0.7–1.1	51–64	1.1–29.2	5–7	^21^
Stick insects (Phasmatodea: Timematidae)	d	12–14.9	0.5–0.9	36–75	7–19	4–8	^18^
Snow fleas (Mecoptera: Boreidae)	d	6.2–7.4	0.7–0.9	106–121	0.9–1.3	11–16	^19^
Scorpion fly (Mecoptera: Panorpidae)	d	1.4–19.0	0.7–1.9	38.6–1600	0.6–23.4	5–160	^23^
Parasitoid wasps (Hymenoptera)	d	5.0–74.0	0.2–0.9	8–163	0.01–10.9	1–17	^22^
Lacewings (Neuroptera: Chrysopidae)	d	9.3–19.0	0.5–1.0	39–62	0.5–56	4.0–6.3	^20^

**Table 2:** Kinematic parameters of jumping conditions in the marsh beetle *Scirtes hemisphaericus* (Coleoptera: Scirtidae), average values in brackets.

Jump mode	Take-off time (ms)	Velocity (m s^−1^)	Acceleration (m s^−2^)	Kinetic energy (× 10^–6^ J)	g-force
All modes combined	2.7–8.4 (4.1)	0.4–1.9 (1.3)	139–1536 (743)	0.2–5.4 (2.9)	14–156 (75)
Two legs	2.7–5.4 (3.5)	1.0–1.8 (1.5)	718–1536 (941)	1.7–5.1 (3.8)	73–156 (95)
One legs	3.0–6.3 (4.4)	0.5–1.7 (1.0)	194–768 (443)	0.5–4.7 (1.9)	19–78 (45)
Flight (wings opened)	3.3–8.4 (5.0)	0.4–1.9 (1.0)	139–1203 (614)	0.2–5.4 (2.2)	14–122 (62)

The power output (P, W kg^−1^) of a single jumping leg was calculated using the formula:

$$P = \frac{{K_{e} }}{{\Delta t \cdot 2m_{l} }}$$

where, K_e_ is the kinetic energy (J) of the jump, Δt is the time interval (s) of the rapid increasing of kinetic energy value (determined from the high-speed videography), 2 m_l_ is the mass of two jumping legs (kg). The calculated power output of the jumping leg is ranged from 3.5 × 10^3^ to 9.6 × 10^3^ W kg^−1^ (3.5–9.6 W g^−1^) and the average value is 6.98 × 10^3^ W kg^−1^ (SD = 1.64 × 10^3^). However the instantaneous power could be much higher.

## Discussion

Rapid lifting up the body into air in the majority of jumping representatives of insects is performed by legs. The corresponding leg movements usually involve flexion of tibiae followed by their rapid extension. It can be accompanied by additional power amplification or without it. In the first case that is called the ‘spring-actuated mechanism' (known also as a ‘catapult mechanism’) the muscle contraction is reinforced by additional energy typically stored in elastic or/and resilient structures^1^, whereas in the second case the jump is powered by the direct muscle contraction alone. The extensive studies of jumping insects suggest the spring-actuated jumping mechanism for the fleas (Siphonaptera)^3,4^, grasshoppers and locusts (Orthoptera)^5,6,9–11,29,30^, beetles Chrysomelidae and Curculionidae (Coleoptera)^25,26^, shore bugs Saldidae (Heteroptera)^17^, planthoppers Issidae (Auchenorrhyncha)^16^, froghoppers Cercopoidea (Auchenorrhyncha)^14,31^, leafhoppers (Auchenorrhyncha)^12,13,15^, and pygmy mole crickets Tridactylidae (Orthoptera)^32^. Jump by the direct muscle contraction is supposed for the bush crickets Tettigoniidae (Orthoptera)^8^, stick insects Timematidae (Phasmatodea)^18^, lacewings Chrysopidae (Neuropetra)^20^, caddies flies (Trichoptera)^21^, parasitoid wasps Pteromalidae, Braconidae, Figitidae, Ichneumonidae (Hymenoptera)^22^, snow fleas Boreidae (Mecoptera)^19^, and scorpion flies Panorpidae (Mecoptera)^23^.

The jumps based on the spring-actuated mechanism and by direct muscle contraction differ by their kinematic parameters (Table 1). One of the most representative parameters, take-off time, in the insects with catapult mechanism ranges from 0.7 to 8.4 ms versus 1.4 to 74.0 ms in insects with the jump by direct muscle contraction. The same holds for the velocity (0.4–5.5 m s^−1^ vs. 0.2–2.2 m s^−1^) and acceleration (100–7051 m s^−2^ vs. 8–1600 m s^−2^), respectively. One of the obvious reasons for the difference in kinematic parameters is the limitations imposed by the direct muscle contraction. It is known that direct muscle contraction in insects allows them to achieve the maximum specific power output of a joint^33–36^ at about 100 W kg^−1^.

The values of the kinematics parameters of marsh beetle's jumping (Table 1) are in good agreement with those for insects using power amplification for the jumping process. The time required to fully extend the tibia reaches up to 2.4 ms (Fig. 5) in our best records. It significantly exceeds the temporal limitations known for insect muscle contractions^1,35,37,38^. The calculated joint power output value of 6.98 × 10^3^ W kg^−1^ significantly exceeds the maximum of 100 W kg^−1^ that can be achieved by the direct muscle contraction. These data convincingly indicate that such values require the use of additional potential energy.

The energy can be stored in deformation, e.g. stretching or compressing of elastic or/and resilient structures until the energy is released. In the leg-powered jumping insects, such reverse deformation occurs instantaneously in spring-actuated mechanism. Thus, for the functioning of the spring-actuated mechanism, the presence of such specific elastic structures is necessary to preserve elastic strain energy necessary for the jump performance. In insects, as a rule, such structures contain a rubber-like protein, resilin (e.g.^39–41^). Resilin has been revealed in many jumping insects^1^, e.g. in the semi-lunar processes of the hind femoro-tibial joint of locusts^9,30^, the pleural arch of froghoppers and planthoppers^15,31,42^, special patches in the metathorax of fleas^4^ and the extensor ligament of the hind legs of flea beetles and weevils^25,26^. In marsh beetles, resilin is found in the extensor ligament connecting the extensor muscle and the base of the tibia (Fig. 4F–I). Thus, the ligament is an integral part of the jumping apparatus and is directly involved in its functioning. It can be assumed that the deformation of the ligament and, accordingly, the preservation of elastic strain energy occur, when the ligament is stretched by the extensor muscle. In this case, the tibia should be pressed against the femur, in order to provide a forward movement, when extending and pushing off from the substrate surface.

Since the extensor muscle fibre bundles are much stronger than those of the flexor muscle, it is necessary to avoid premature extension of the tibia, when stretching the ligament. For this purpose, there are several latching mechanisms found in insects (^43^; see also more broad discussion about power amplification and latch-mediated springs in^44,45^) such as: (1) passive latching mechanisms^14^, (2) active latches^1,4^ and (3) co-contractions of antagonistic muscles with corresponding leverage^1,5,25,26,29,46^. It is assumed that in *Scirtes hemisphaericus* the conical projection of the TFS (Fig. 4A,C–E) can serve as an active latch and prevent premature extension of the tibia. There are several evidences in favour of this idea: (1) the shape of the conical projection, which exactly corresponds to the impression and angular ridge near the lateral projection of the tibia (Fig. 4D), (2) the location of the conical projection in the corresponding impression on the tibia (when the tibia fully flexed) in such a way that the lateral protrusion abuts directly against the conical projection (Fig. 4D). While the conical projection is in this position and the lateral projection of the tibia abuts against it, the latter cannot be extended. In order to unlock and perform extension of tibia, it is necessary to pull out the conical projection from the corresponding socket by pulling it back by the short contraction of the flexor muscle (Fig. 4A).

**Figure 8:**
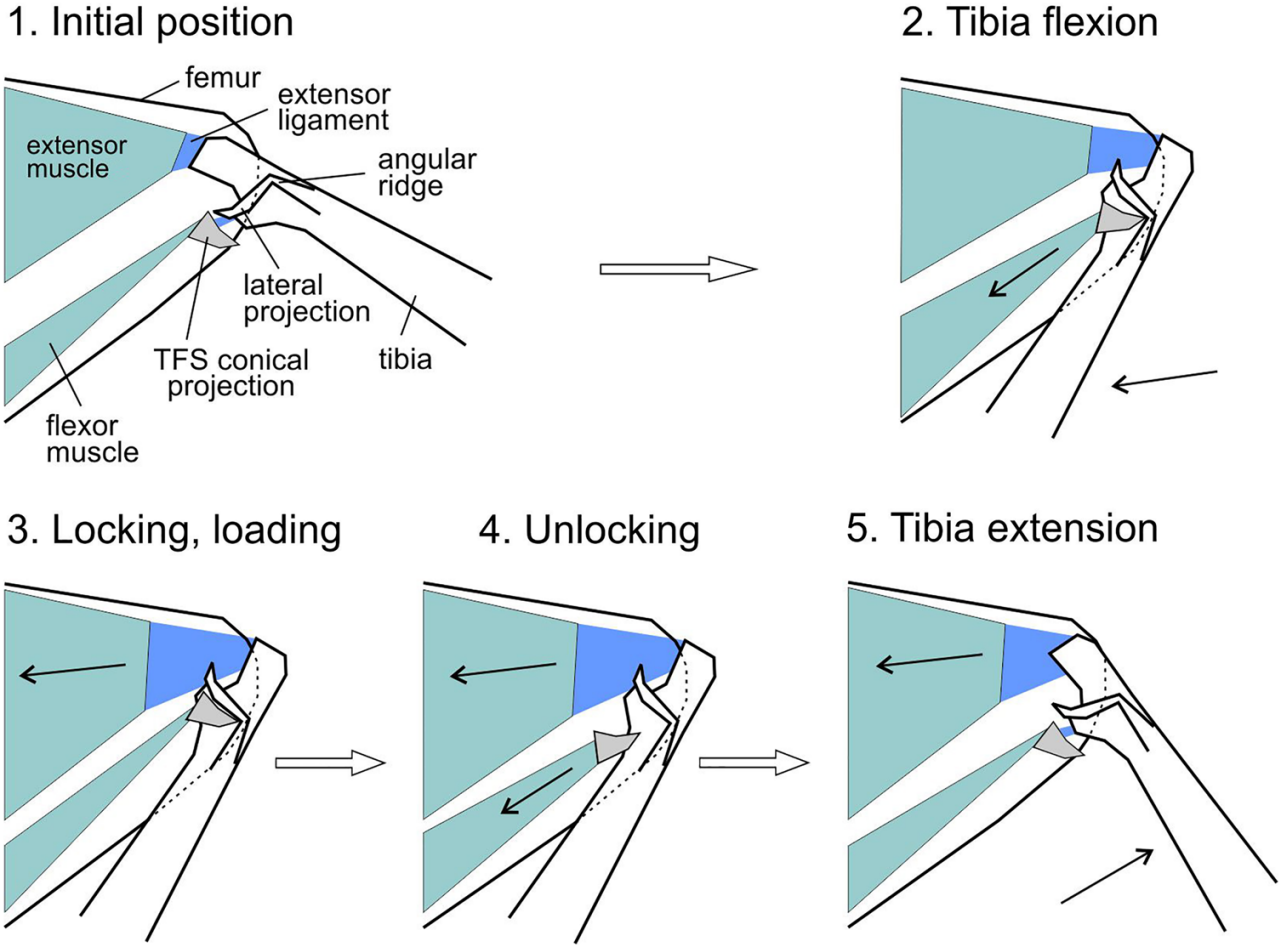
Scheme of the jumping process depicting its main phases. *TFS* tibial flexor sclerite. Detailed description of the jumping according to phases see in the “Discussion”. White arrows show the direction of phases; black arrows inside the muscles show the direction of muscle contraction, black arrows at the tibia in the phase 2 and 5 show the direction of tibial movement, flexion and extension respectively.

Based on the above-mentioned, we propose the following functional mechanism of jumping in marsh beetles (Fig. 8). (1) Tibia is flexed completely by the flexor muscle, extensor muscle is relaxed, resilin-bearing extensor ligament is partially stretched (Fig. 8 phase 2). (2) Conical projection of the TFS, inserted into angular projection and impression at the lateral side of the tibial base, locks the tibia in a fully flexed position (Figs. 4D, 8 phase 3). (3) Extensor muscle contracts and stretches the extensor ligament further. (4) Prior to jump, the flexor muscle shortly contracts and pulls the conical projection slightly backward. The conical projection goes out from the angular impression (angular socket) and unlock the tibia (Figs. 4A, 8 phase 4). (5) The preloaded and stretched extensor ligament, accompanied by the extensor muscle, starts to contract rapidly and pulls the tibia that in turn extends and moves the body up (Figs. 4E, 8 phase 5).

The femoro-tibial joint of the hind jumping legs of the marsh beetles morphologically strongly differs from that of other jumping beetles. Despite of some lack of broad comparative morphological studies on this topic, one may discuss the generalized structure of the femoro-tibial joint^47,48^ as that having such specific elements as a pair of symmetric femoral condyles (convex, circular structure of the toroid shape) and a pair of tibial concavities with the corresponding complementary shape. The femoral condyle inserts into the tibial concavity and is bent by the ligament, through which the single axis of rotation runs. In the jumping marsh beetles, both lateral and medial triangular invaginations (Figs. 2C,D, 3C,E,F) of the femur serve as sockets for the tibial projections. The frontal protruding edge of the dorsal part of the invaginations serves as a support for the corresponding small depressions situated right above the tibial projections. The axis of rotation of the joint passes through these projections and depressions (Fig. 3B). When the tibia is flexing to the femur, the both projections of the tibia enter the corresponding invaginations on the femur and place themselves transverse to the invaginations (Fig. 4B). The anterior edge of the dorsal part of the invaginations is larger, thicker and more prominent (Figs. 2C,D, 3E,F), since it withstands most of the load during flexion of the tibia and contraction of the extensor muscle. The functions of the both projections of the tibia are supposed to be as follows: (1) to direct the movement of the tibia during flexion and to prevent its displacement to the sides, (2) to be a mechanical support for the femoro-tibial joint, (3) the lateral projection is the part of the locking mechanism. The asymmetry of the tibial projections (Fig. 3G) is due to the fact that the ventral side of the lateral projection and the impression behind it also serve as a socket for the conical projection.

Jumping beetles are characterized by the presence of the sclerotized metafemoral extensor tendon (MET) as a structural part of the jumping apparatus, which is connected to the tibia by the extensor ligament. This was previously found in leaf beetles Chrysomelidae (subfamilies Bruchinae and Galerucinae), weevils Curculionidae (subfamilies Curculioninae, Erirhininae and Ceutorhynchinae) and jewel beetles Buprestidae (subfamilies Agrilinae and Trachyinae)^24–26^. The MET serves as an attachment site for the extensor muscle, increasing the surface area for attachment and possibly providing additional strength to the femoro-tibial joint system^25,26^. In contrast, marsh beetles lack MET, yet like other jumping beetles accumulate the additional elastic strain energy for the spring-actuated mechanism in the extensor ligament stretching by the extensor muscle. Unlike marsh beetles, the co-contraction of antagonistic muscles (extensor and flexor) and a leverage to overpower the stronger extensor muscle by the weaker flexor have been previously discovered for flea beetles and weevils as the mechanism preventing premature extension of the tibia^25,26^. In contrast to other jumping beetles, marsh beetles utilize for this purpose the active latching mechanism with an active latch system. Supposedly functionally similar, however not fully understood and explored, presumable active latches are supposed for the jumping mechanism in fleas^3,49–51^. Active latch mechanism is also known for the trap-jaws of *Odontomachus* ants^52–54^. However, in these cases supposedly a specialized small muscle serves as a trigger to unlock the latch. In marsh beetles, a specialized trigger muscle has not been found, and its role is played by the flexor muscle that makes this latching mechanism simpler and presumably more reliable. Based on the examination of the additional material on the genera *Scirtes* and *Ora* (*Scirtes orbicularis* Panz., *S. brunneus* Zwick, *S. kodadai* Yosh. et Ruta, *S. schawalleri* Yosh. et Ruta, *S. teruhusai* Yosh. et Ruta) and literature data on the morphology of these genera^55–58^ one may suggest the principal similarity in structure of their hind jumping legs and, hence the jumping mechanism.

All in all, the marsh beetles evolved their own jumping mechanism lacking MET and utilize an active latching system discovered in beetles for the first time and that can be considered as a good example demonstrating morphological and functional flexibility of hind legs. Finally, this study shows a variety of pathways of insect locomotory apparatus in solving similar evolutionary challenges.

## Material and methods

### Animals

The jumping mechanism, structure of the jumping apparatus, jumping performance, and kinematics were examined and analysed in the marsh beetle *Scirtes hemisphaericus* (Linnaeus, 1767) (Coleoptera: Scirtidae) (Fig. 1). The beetles were collected by a sweeping net from the vegetation at the pond shore of the University campus of the CAU (Kiel, Germany) during the summer of 2020. The beetle specimen of *S. hemisphaericus* for the synchrotron X-ray micro-computed tomography is taken in loan from the collection of the Stuttgart State Museum of Natural History (SMNS, Germany). Additional material from SMNS on the species *Scirtes orbicularis* Panz., *S. brunneus* Zwick, *S. kodadai* Yosh. et Ruta, *S. schawalleri* Yosh. et Ruta, *S. teruhusai* Yosh. et Ruta have also been examined. The hind legs of these species were examined in order to reveal the presence of morphological characteristics specific for jumping legs, namely, 1. swollen metafemur, and 2. >-shape structures (lateral and medial invaginations, Fig. 2).

### Material preparation

All material preparation was carried out under a stereomicroscope (MZ7.5, Leica Microsystems GmbH, Germany). Freshly anesthetized beetle individuals and those fixed in 70% ethanol were examined. Fine needles and razor blades were used for dissections, which were carried out under distilled water or 70% ethanol.

### High-speed videography, kinematics parameters and power output calculations

Jumping performance was examined by using a high-speed video camera (Photron Fastcam-1024PCI, Photron USA Inc., San Diego, U.S.A.) combined with a stereomicroscope Leica MZ 12.5 (Leica Microsystems GmbH, Wetzlar, Germany). Beetles were observed in a transparent plastic cube of 2 × 2 cm; their jumps were recorded without stimulation at a rate of 3000 fps. In total, 25 individuals of *S. hemisphaericus* were recorded by high-speed videography. This corresponds to 25 high-speed videorecording, with 20 of them being chosen for the calculation of kinematic parameters (Tables 1 and 2) by using Tracker ver. 5.1.5, 2020 software^59^, https://physlets.org/tracker/). For the calculation of the kinematic parameters, five freshly anaesthetized beetles were weighed separately each on an ultramicrobalances (Sartorius MSE2.7S, Sartorius Lab Instruments GmbH & Co. KG, Göttingen, Germany). The average mass of a single individual was found to be 2.95 × 10^–6^ kg (2.95 mg) (± 1 × 10^–7^ kg, range 2.37 × 10^–6^–4.18 × 10^–6^ kg [2.37–4.18 mg]). The length of beetles ranged from 0.003 m to 0.0036 m (3.0–3.6 mm). The average length of a single individual was 0.0033 m (3.3 mm). For the calculation of the jumping leg power output, the mass of a jumping leg were measured in the following way. Ten fresh hindlegs were separated from the body and immediately weighed together using an ultramicrobalances, the obtained mass was then divided by 10, and the average mass of a leg was found to be 1 × 10^–7^ kg (0.1 mg). The other values were taken from the kinematic parameters calculated by the Tracker software. The contributions of potential energy change and of the work to be done against the air resistance (or drag force) was calculated in the following way: potential energy during lift-off was calculated as m × g × h = 2.9 × 10^–8^ J which is just about 1% of kinetic energy. The contribution of air drug ($F_{d} = 0.5C_{d} \rho V^{2} A,$ where C_d_ = 0.42 is a drag coefficient for half-sphere, ρ is air density, V is animal velocity, and A is a cross sectional area) is even less and the calculated value was ~ 2 × 10^–9^ J. So that both effects were further neglected at the calculation of leg power output. The leg power output was calculated only for the beetle jumping with two legs only (not with one leg) with fully closed wings. The values obtained using the formula (see below in the text) was calculated for a single jumping leg. Image processing and preparation for publishing were carried out by Adobe Photoshop® CE5 EXD (Adobe Systems Inc., U.S.A.) and CorelDraw® X5 (Corel Corp., Canada).

### Scanning electron microscopy (SEM)

Hind legs from ethanol-preserved specimens were removed from the body, dissected with a razor blade in sagittal plane, air dried at a room temperature for at least 24 h. Then dissected samples of femurs and tibiae were glued onto aluminium SEM stubs, coated with gold–palladium using a Leica EM SCD500 sputter coater (Leica Microsystems GmbH, Wetzlar, Germany), and examined with a Hitachi S4800 (Hitachi High-Technologies Corp., Japan) scanning electron microscope at 3 kV.

### Light microscopy

The light microscopy images from dissected and non-dissected samples of the hind legs, mounted on the glass slides in glycerine, were taken by a light microscope Zeiss Axioplan (Carl Zeiss Microscopy GmbH, Oberkochen, Germany).

### Confocal laser scanning microscopy (CLSM)

The dissected and non-dissected samples of the hind legs were mounted on the glass slides in glycerine and visualized with a confocal laser scanning microscope Zeiss LSM 700 (Carl Zeiss MicroImaging GmbH, Oberkochen, Germany) equipped with three stable solid-state lasers. The detection of resilin was visualized using the 405 nm laser line and a bandpass emission filter transmitting 420–480 nm. To visualize autofluorescences from chitin and muscles the 488 and 639 nm laser lines were used in combination with longpass emission filters transmitting light with wavelengths 493–550 nm and 644–800 nm, respectively.

### Synchrotron X-ray micro-computed tomography (SR-µCT)

The hind legs from the dry museum specimen were glued onto the tip of a plastic stub (1.2 cm long; 3.0 mm in diameter). SR-µCT was carried out in the ID19 beamline at the European Synchrotron Radiation Facility (ESRF, Grenoble; experiment LS-2342) at 19 keV (wavelength of 8 × 10^–11^ m) and an effective detector pixel size of 0.65 µm with a corresponding field of view of 1.43 × 1.43 mm; 6000 projections were recorded over the 180° rotation. The detector-to-sample distance was 12 mm. For 3D reconstruction, we used the graphic segmentation tool software Amira^®^ 6.0 (FEI Company, Visage Imaging, Germany) and the volume graphics visualization Drishti 2.5.1^60^.

## Supplementary Information

Supplementary Video 1.

Supplementary Video 2.

Supplementary Video 3.

Supplementary Legends.

## Data Availability

The datasets used and/or analysed during the current study available from the corresponding author on reasonable request.
