# Supplementary material for: Jumping mechanism in the marsh beetles (Coleoptera: Scirtidae)
Source: Sci Rep. 2022 Sep 22;12:15834. doi: 10.1038/s41598-022-20119-5 (PMC9500066; doi:10.1038/s41598-022-20119-5)
Supplement: Supplementary file 4 — Supplementary Legends. [file 41598_2022_20119_MOESM4_ESM.docx]

**Supplement Information**

To the paper of Nadein, K., Kovalev, A., Gorb, S. ‘Jumping mechanism in the marsh beetles (Coleoptera: Scirtidae)’. Scientific Reports.

Legends to movies

SI Movie 1. Marsh beetle *Scirtes hemisphaericus*. Jump is elicited by two hind legs, 3000 frames s^-1^.

SI Movie 2. Marsh beetle *Scirtes hemisphaericus*. Jump is elicited by one hind legs, 3000 frames s^-1^.

SI Movie 3. Marsh beetle *Scirtes hemisphaericus*. Jump is elicited by two hind legs with opened wings, 3000 frames s^-1^.
